# Positive Feedbacks Enhance Macroalgal Resilience on Degraded Coral Reefs

**DOI:** 10.1371/journal.pone.0155049

**Published:** 2016-05-17

**Authors:** Claire L. A. Dell, Guilherme O. Longo, Mark E. Hay

**Affiliations:** School of Biology and Aquatic Chemical Ecology Centre, Georgia Institute of Technology, Atlanta, Georgia, United States of America; Dauphin Island Sea Lab, UNITED STATES

## Abstract

Many reefs have shifted from coral and fish dominated habitats to less productive macroalgal dominated habitats, and current research is investigating means of reversing this phase shift. In the tropical Pacific, overfished reefs with inadequate herbivory can become dominated by the brown alga *Sargassum polycystum*. This alga suppresses recruitment and survival of corals and fishes, thus limiting the potential for reef recovery. Here we investigate the mechanisms that reinforce *S*. *polycystum* dominance and show that in addition to negatively affecting other species, this species acts in a self-reinforcing manner, positively promoting survival and growth of conspecifics. We found that survival and growth of both recruit-sized and mature *S*. *polycystum* fronds were higher within *Sargassum* beds than outside the beds and these results were found in both protected and fished reefs. Much of this benefit resulted from reduced herbivory within the *Sargassum* beds, but adult fronds also grew ~50% more within the beds even when herbivory did not appear to be occurring, suggesting some physiological advantage despite the intraspecific crowding. Thus via positive feedbacks, *S*. *polycystum* enhances its own growth and resistance to herbivores, facilitating its dominance (perhaps also expansion) and thus its resilience on degraded reefs. This may be a key feedback mechanism suppressing the recovery of coral communities in reefs dominated by macroalgal beds.

## Introduction

Coral reefs world-wide have been losing coral cover and gaining macroalgal cover [1,2]. This is an undesirable shift in the community composition because macroalgal dominated reefs lose topographic complexity, support fewer other species, and provide fewer ecosystem services than coral dominated reefs [3]. Macroalgal dominance is also a key factor preventing the recovery of corals [4,5] and this may be due to the establishment of feedback mechanisms that reinforce the dominance of the new macroalgal dominated state [1,6]. There is thus a need for greater understanding of feedbacks potentially enforcing the macroalgal dominated state and hindering coral recovery, upon which a host of other species depend [2,6].

To date, known feedback mechanisms function by negatively affecting corals and fishes. Macroalgae reduce growth of coral recruits [7] and adults [8], negatively impact fecundity [9], and increase coral mortality via allelopathy [10,11] and by vectoring coral disease [12]. Macroalgae also deter recruitment of both corals and fishes [5,13]. In addition, rapid macroalgal growth can overtake the grazing ability of herbivorous fishes [14,15] leading to the formation of dense macroalgal stands that can hinder browsing fishes [16]. Furthermore, mature algae may be less palatable than young recruits [17], so if not suppressed at this stage algae can develop into sizes or morphologies that suppress grazing [18,19]. This may further promote the persistence of mature macroalgal beds. If herbivores recover to adequate densities and functional diversities, they can remove seaweeds, facilitating reef recovery [20, 21, 22], but algal properties preventing or delaying such recoveries are not well understood.

However all of these mechanisms focus on the impact macroalgae have on other species; the effect macroalgae have on their own species is relatively un-investigated. Our aims were therefore: 1) to investigate the processes controlling macroalgal populations, 2) to understand the impacts of these processes on recruit-sized versus mature-sized algal ramets, 3) to evaluate how these processes varied between areas of different community compositions (fished areas dominated by macroalgae versus protected reefs dominated by corals), and 4) to determine whether these processes might generate feedback mechanisms that facilitate macroalgal persistence. Specifically, we tested for the effects of herbivory and the presence of conspecifics on the survival and growth of mature-sized and recruit-sized macroalgae, and used comparisons between coral dominated marine protected areas (MPAs) and adjacent fished reefs (non-MPAs dominated by macroalgae) in Fiji to assess whether the influence of these factors varied between these habitats. Additionally, because population divergence has been documented to occur within a few generations when selection is strong [23,24], we accounted for the possibility of rapid evolution in this system by examining growth and survival by algal origin (MPA vs. non-MPA) and also by analysing microsatellite loci to determine if populations were differentiated by site.

## Methods

### Ethics statement

This work was conducted in accordance with the animal care guidelines of the Georgia Institute of Technology’s Institutional Animal Care and Use Committee (IACUC). Approval was granted prior to commencement of research (Permit A12085). Permission to perform this research was granted by the Korolevu-i-Wai environmental committee and district elders and by the Fijian Government.

### Study site and species

**Fig 1:**
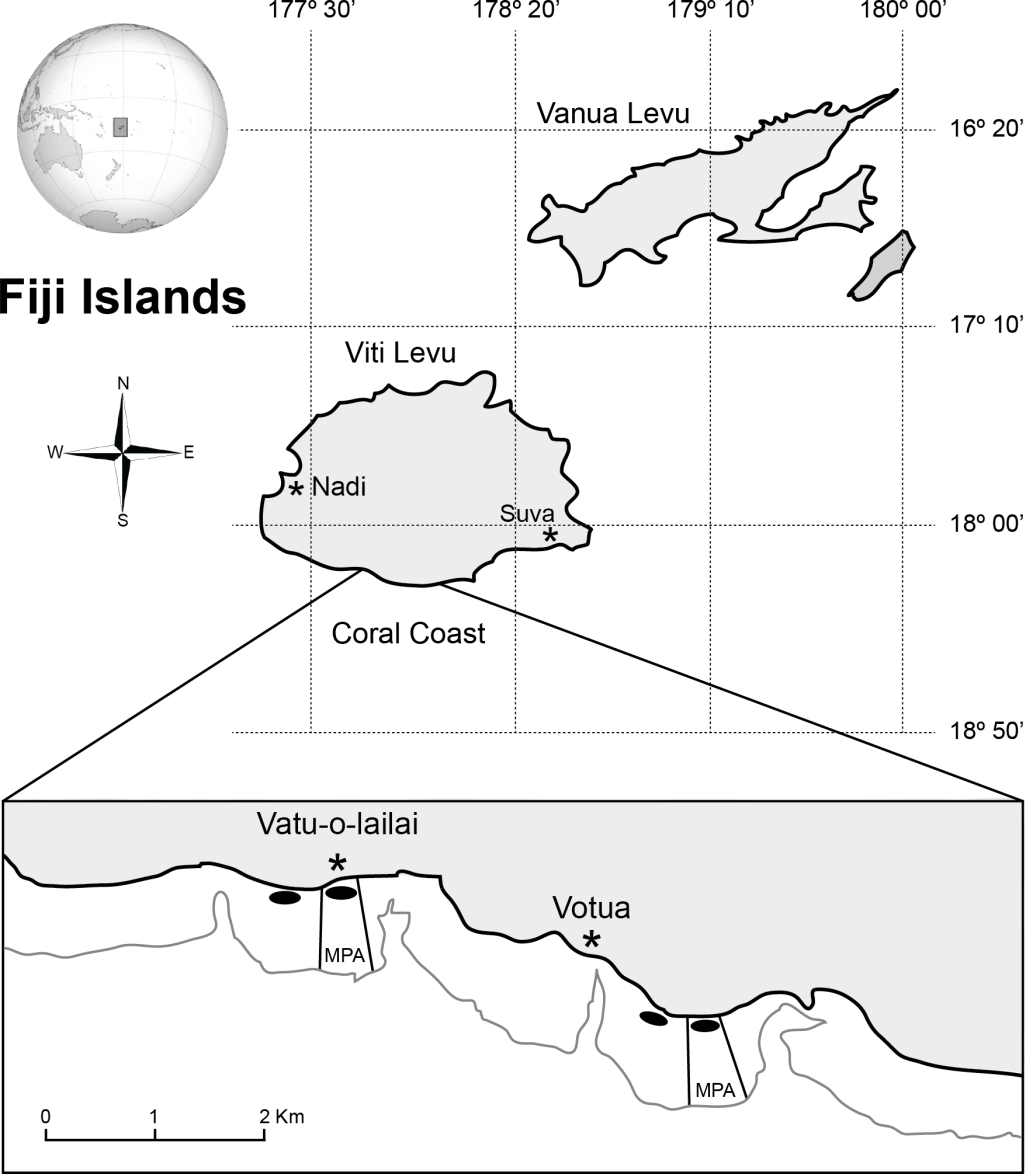
Locations of villages, MPAs, sample collections, and experiments. Ovals represent the area in which samples were collected for microsatellite analysis and where experiments involving caging of mature *S*. *polycystum* fronds and transplanting of recruit-sized ramets were conducted. Experiments caging recruit-sized ramets and transplanting adults were run slightly seaward. A portion of the figure modified from [26, 27].

This study was conducted between January and May in 2013 and 2015 on the coral coast of Fiji’s main island, Viti Levu, in the villages of Votua and Vatu-o-lailai (18°12’32S, 177°42’00E and 18°12’13S, 177°41’29E respectively; Fig 1). These villages are ~3km apart and each has jurisdiction over their stretch of reef flat; a habitat ranging between ~1.5 and 3m deep at high tide and between ~0 and 1.5m deep at low tide. In 2002, these villages established small areas (0.8km^2^ in Votua and 0.5 km^2^ in Vatu-o-lailai; Fig 1) as no-take MPAs [25]. Though MPA and non-MPA areas were initially similar in coral and macroalgal cover (33–42% macroalgal cover; 3–12% coral cover [25]), MPAs now differ significantly from the adjacent non-MPAs in benthic cover and fish diversity and abundance. MPAs now have ~56% live coral cover on hard substrate, ~2% macroalgal cover, ~8 fold higher biomass of herbivorous fishes, and higher recruitment of both fishes and corals than the non-MPAs [5,22]. Meanwhile the non-MPAs have lower fish biomass, 5–16% live coral cover on hard substrates and 51–92% macroalgal cover, the majority of which is comprised by Phaeophytes (primarily *Sargassum polycystum* C. Agardh [22]). In the MPAs, macroalgal cover is restricted to the shallowest, most shoreward areas (where access by herbivorous fishes appears limited), whereas macroalgal cover in the non-MPAs extends throughout the habitat. Thus, over distances of only a few hundred metres, there are dramatic differences in community composition that may impact the efficacy of factors controlling macroalgal populations, without the confounding factors of great differences in space or time.

We used *Sargassum polycystum* as a study organism because it is often the most conspicuous macroalgal species on degraded Pacific reefs and can grow to dominate large areas [22,28–30]. On reefs lacking adequate herbivory, *S*. *polycystum* can reach 8.55 kg wet weight per square metre [28] and its odour can suppress both fish and coral recruitment [5], potentially limiting reef recovery. In Fiji, perennial holdfasts start regenerating in December and by the end of its growing season in June, *S*. *polycystum* commonly dominates large expanses of the unprotected reef flats [22,29]. Around this time it may reproduce sexually via spores that disperse only one to three metres [31], suggesting the potential for reduced connectivity between even nearby sites. After June, *S*. *polycystum* senesces leaving the perennial rhizomes sheltered within the reef structure. Populations in our study area will have undergone about 10 generations since MPA establishment, which has been shown to be adequate time for population differentiation among some species if selection is strong [24,32].

### Effect of habitat and origin on the survival and growth of mature *S*. *polycystum* fronds

The dearth of *S*. *polycystum* in the MPAs and its high abundance in the non-MPAs could be due to differing physical conditions in those locations. To investigate the role of physical conditions and to test whether *S*. *polycystum* in these areas was acclimatising to the different local conditions, a reciprocal transplant experiment was performed between the MPAs and non-MPAs at two villages to measure survival and growth of mature *S*. *polycystum* as a function of origin (from the MPA or non-MPA) and habitat (placed in the MPA or non-MPA) when the fronds were protected from herbivory in cages.

The uppermost 15 centimetres of a *S*. *polycystum* frond was collected from 40 separate holdfasts in the MPA and 40 in the non-MPA of the villages of both Votua and Vatu-o-lailai. To minimise the likelihood of collecting multiple fronds from a single clone, the holdfasts were separated by at least two metres. The lowest five centimetres of each frond were defoliated, the fronds were then blotted dry with paper towels and weighed to the nearest 0.1g. The top of the defoliated section was marked by piercing the thallus with a needle and tying a thread at this 5cm point to set a standard from which to measure growth in length. One strand of *S*. *polycystum* from the MPA and one from the non-MPA were affixed 20cm apart in the centre of a 50cm piece of 3-strand rope. The lowest 5cm of each algal stipe was threaded through the rope to anchor the strand in place. Four ropes were affixed in each of five cages (dimensions 1m x 1m x 0.8m constructed of 1cm mesh) by the two 10cm end sections of each rope so that the rope’s centre, holding the algae, was raised a few centimetres above the substrate. Five cages were anchored at a depth of ~1.2m at low tide in both each MPA and non-MPA so that cages at each location were separated by a minimum of two metres. After one month, the length (from the threaded point) and mass of each frond were measured to assess growth.

Change in length was measured in centimetres after two and four weeks. As mass measurements required removing the fronds from the water, to minimise stress to the organism, change in mass was measured in grams only after four weeks. Because significant effects were the same in each of these data sets, only results from height change at week four are reported. A mean change in length was calculated separately for the MPA and non-MPA adults in each cage, yielding an n = 5 for each location. Within each independent cage, we calculated the mean growth of MPA origin fronds, the mean growth of non-MPA fronds, and used the difference between these values in paired t-tests run separately for each location testing the effect of origin on growth over the four weeks. These difference scores were normally distributed.

To investigate the effect of habitat (MPA or non-MPA) on growth, a Mann-Whitney U test compared MPA originated fronds transplanted into both habitats; the same was done for non-MPA originated fronds. All analyses were conducted in SPSS version 16.0 with α adjusted to α = 0.025 to account for the multiple contrasts.

### Effects of habitat, origin and herbivory on survival of recruit-sized *S*. *polycystum*

In parallel to the above experiment, we addressed the effects of origin, habitat, and herbivory on survival of recruit-sized *S*. *polycystum*, by performing a reciprocal transplant between Votua’s MPA and non-MPA.

Small *S*. *polycystum* ramets ~1cm long (range between 0.5cm and 1.5cm) were collected from both the MPA and non-MPA using a nail and hammer so that a small piece of bedrock remained attached to each alga’s holdfast, allowing four ramets from either the MPA or the non-MPA to be affixed to ~25cm^2^ tiles by attaching the rock pieces using aquarium glue (Ecotech Marine, USA). The ramets were selected so that the four on each tile were of equal origin and size and were arranged in a square pattern 1cm distance from each other. The tiles were placed in coolers, containing a few centimetres of seawater and left for 12 hours in the shade to allow the glue to set before moving the tiles to the reef. The tiles were paired so the MPA and non-MPA ramets were of equal size and one tile of each was affixed in a cage so they were 30cm from each other.

These cages were either complete, so the ramets would be protected from fish grazing, or open-sided, so the ramets would be exposed to fish grazing. The open cages lacked the 2 walls parallel to the current direction so that fish access was permitted, while cage effects on flow and shading would be as similar as possible between treatments. The base of each cage was 0.75m x 0.75m, the height was 0.75m and the mesh size was 1cm^2^ thus excluding all but the smallest fishes and invertebrates. Ten replicates of each treatment were distributed in Votua’s MPA and 10 in Votua’s non-MPA so that the complete and open cages were paired and the cages in each pair were about one metre apart, while the distance between pairs was ≥ two metres. These cages were distributed ~25 to 50m from shore at a depth of ~1 to 1.5m at low tide.

The experiment was established mid- January 2013, ran for 4 months (112 days), and was checked for ramet mortality every 3 days for the first month and then every week. If an alga was missing but the stone remained, this was noted as mortality. If the stone was also missing this could have been due to failure of the glue, dislodgement by turbulence, or some unknown agent, so we recorded these as ‘lost’ and excluded them from analysis. Only ten ramets (3.1%) were lost which reduced the total number of ramets in the experiment from 320 to 310.

Despite running for four months and being checked at intervals of 3–7 days throughout this period, we could detect no growth in this experiment so we report only mean duration of survival. Duration of survival was calculated as the average number of days survived by the four MPA ramets and by the four non-MPA ramets in each cage, giving n = 10 for each treatment in each habitat. Difference scores (mean survival duration for MPA versus non-MPA sub-samples in each replicate) were normally distributed (p≥0.200; Shapiro-Wilk) so the effect of origin was analysed by paired t-test run separately for each treatment in each location.

Comparisons of the two treatments (caged or grazed) were performed by independent samples t-tests as all datasets satisfied the assumptions of normality and homogeneity of variance or were successfully log2 transformed to do so. This analysis was run separately for each origin (MPA and non-MPA) in each habitat. As data were analysed twice, we applied the Bonferroni correction with α = 0.025 and ran analyses using SPSS version 16.0.

### Effect of conspecifics on survival and growth of mature fronds

To assess whether conspecific density might facilitate the survival and growth of mature fronds, we transplanted mature fronds into the centre of *Sargassum* beds and into nearby exposed habitats where they were isolated from others. Growth and duration of survival were measured over a two week period. Due to logistical constraints, this experiment was only conducted in Votua’s non-MPA where *Sargassum* beds were extensive and thus many separate patches were available for use.

Eight 10cm fronds of *S*. *polycystum* were removed from the centre of one holdfast, assuring genetic uniformity. Four were threaded through a three-strand rope (secured 5cm apart and 10cm from each end of the rope), and returned to the centre of the *Sargassum* bed (crowded condition) ~75m from shore at a depth of ~1m at low tide. The other four were threaded through a separate rope and tied in an area devoid of *Sargassum* two to four metres away (isolated condition). The ends of the rope were tied to the substrate to hold the rope in place. Twenty such rope pairs were set up with a total of 80 *S*. *polycystum* pieces in each of the crowded and isolated treatments. After two weeks the ropes were collected, the number of remaining fronds was counted and their length was measured. The initial length was subtracted from the final so that fronds that had been grazed in excess of growth were recorded as negative change. An average change in length was calculated per rope from the four fronds in each rope giving an n = 20. This measurement included those that had been grazed and hence included negative change. These data were analysed with a Wilcoxon signed-rank test.

Grazing was either complete (such that none of the frond remained except the section of thallus held between strands of the rope) or absent (such that the entire frond remained including the apical meristem); there were no fronds with portions missing that might indicate partial grazing. Thus, fronds that were grazed or ungrazed could be easily identified. Consequently, we also calculated an estimate of growth from only the ungrazed fronds that had retained their apical meristems and survived the experiment. This permitted a comparison of growth between the crowded and isolated conditions when herbivory appeared to be absent. Once again, the initial length was subtracted from the final length of each ungrazed frond and an average change in length per rope was calculated. As all fronds were completely grazed on four ropes, those pairs were excluded leaving n = 16 in this dataset. Difference scores satisfied the assumption of normality (p = 0.161; Shapiro-Wilk) so data were analysed by a paired t-test. Both analyses were run in SPSS version 16.0 with α = 0.05.

### Effect of conspecifics, origin and habitat on survival and growth of recruit-sized ramets

We investigated the effect of conspecifics on the survival and growth of recruit-sized ramets in conjunction with the effect of origin when ramets were not protected from herbivory. Because *Sargassum* beds in the MPAs only exist near shore and we did not want to confound distance from shore with treatment, we conducted this experiment at a depth of ~0.5m (at low tide) between ~10m to 20m from shore in both Votua and Vatu-o-lailai (Fig 1).

As in the previous experiment that also used recruit-sized ramets, small algal recruits (0.5 to 1.5cm tall) were detached from the substrate so that a small piece of reef substrate remained attached to the alga’s holdfast and these rock pieces were affixed to tiles using Ecotech coral glue. Two MPA and two non-MPA ramets were attached onto each tile in a square pattern 1cm distance from each other. As before, the ramets were chosen so that the four on each tile were of equal size and the tiles were arranged so there was similar size representation of ramets in each treatment. In each location, tiles were placed within established *Sargassum* beds (crowded condition) or placed in open areas (isolated condition) ~2 metres away.

A total of 30 tiles were affixed in the MPA and 30 in the non-MPA within each village, 15 in crowded and 15 in isolated areas. This design ensured there were two origins (MPA or non-MPA) and two density conditions (crowded or isolated) in each of the MPA and non-MPA habitats of both Votua and Vatu-o-lailai.

The tiles were out-planted at the end of February 2013, monitored every 3 days for the first month and then weekly for two subsequent months for mortality and loss. As in the previous tile experiment, if the stone to which the ramet was attached was missing, those individuals were recorded as lost and excluded from subsequent analyses. Of the initial 240 ramets deployed in each village, 16 and 15 individuals were lost (6.7% and 6.2%) from Votua and Vatu-o-lailai, respectively.

At the end of three months, change in height and change in mass were recorded for each ramet. The initial measurement from each ramet was subtracted from its final, meaning the ramets that died were recorded as negative change. An average final height and average final mass were calculated from the two sub-samples (the two MPA and two non-MPA ramets) on each tile giving an n = 15 for each density (isolated/crowded) in each location. These data were analysed by Permutations Analysis of Variance blocked by tile, with origin and density as main effects plus the interaction between the two. This analysis was run separately for each of the four locations using the package lmperm [33] on R version 2.15.3 with α = 0.05. As significant effects were the same for height as for mass data, only results from the height data are shown.

### Microsatellite analysis

To investigate whether differences in *S*. *polycystum* growth and survival correlated with genetic variation among populations, we assessed allelic variation among the four populations (the MPA and non-MPA areas of Votua and Vatu-o-lailai) at five neutral microsatellite loci. Full details of sample collection, sequencing and laboratory methodology for microsatellite analysis are in the supplementary material (S1 Appendix).

The statistic Fst reflects how much of the total population genetic variance is contained in a subpopulation and is thus used as a measure of the genetic divergence between subpopulations [34]. Fst ranges between 0 and 1, where 0 indicates there is no genetic differentiation among populations and 1 indicates complete absence of gene flow among populations. Fst was calculated from the five microsatellites in the four populations using GenePop V4.3 [35].

## Results

### Effect of habitat and origin on the survival and growth of mature *S*. *polycystum* fronds

**Fig 2:**
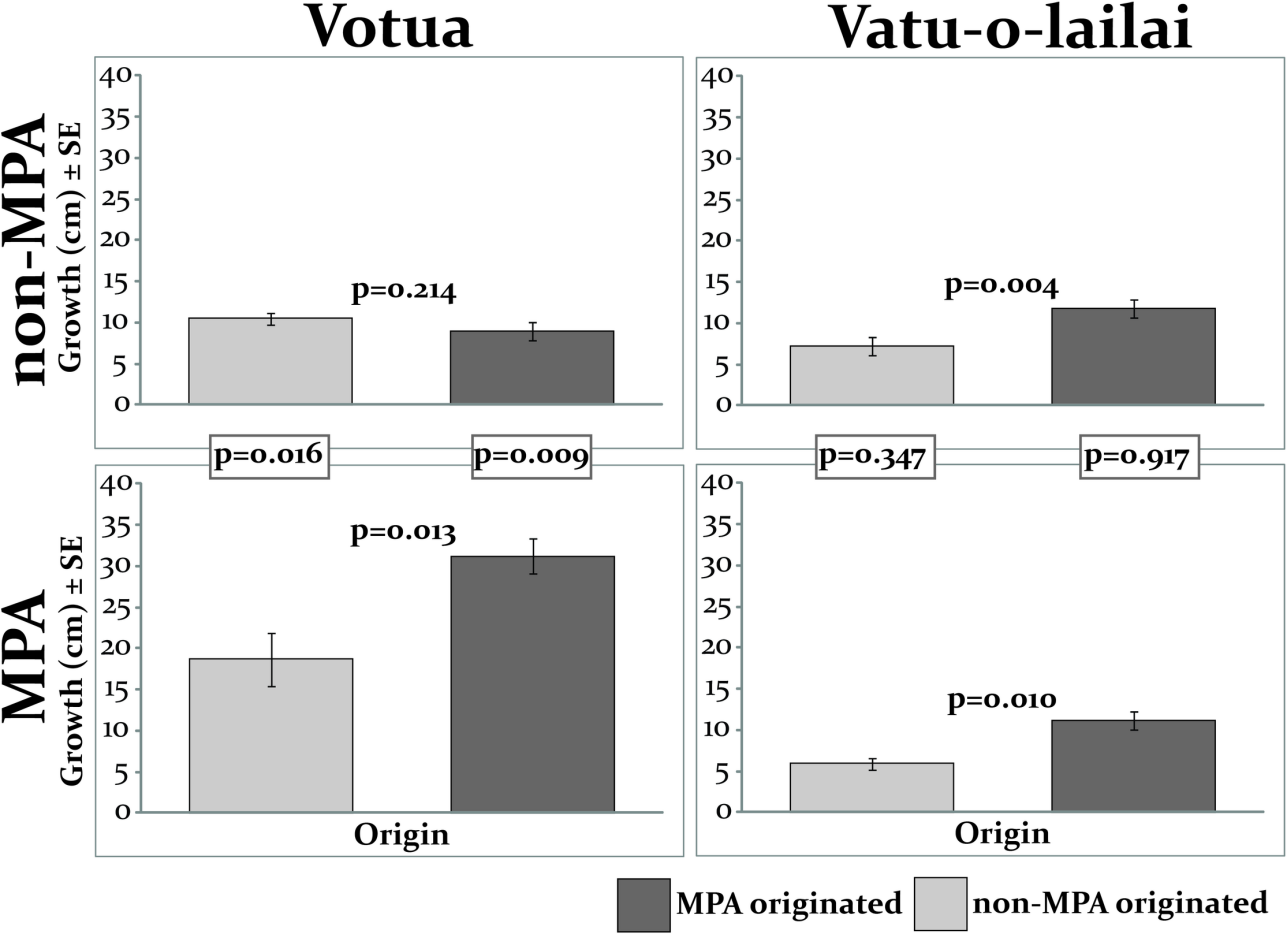
Growth of mature, caged *S*. *polycystum* fronds from MPAs and non-MPAs when reciprocally transplanted p-values above the bars are from paired t-tests assessing the effects of origin. The boxed p-values are from Mann-Whitney U tests assessing the effect of habitat (placement within the MPA or non-MPA) on growth. N = 5 at each location; α = 0.025 to correct for multiple contrasts.

When protected from grazing (i.e., all replicates in this experiment were within herbivore exclusion cages), survival of mature fronds was 100% regardless of location. *S*. *polycystum* originating in the MPA grew significantly more (~40%) than those from the non-MPA in three of the four locations, suggesting a considerable effect of algal origin on growth of mature fronds (p≤0.013; paired t-tests; Fig 2). Conversely in Votua’s non-MPA, the MPA and non-MPA fronds grew at indistinguishable rates (p = 0.214; Fig 2).

An effect of habitat on growth was observed in Votua, where both MPA and non-MPA-originated algae grew a significant 1.8 and 3 times more, respectively, in the MPA than in the non-MPA (p≤0.016; Mann-Whitney U tests) suggesting a strong effect of habitat on growth in this village. In contrast, in Vatu-o-lailai there was no effect of habitat on growth (p>0.347; Fig 2).

### Effects of origin, habitat and herbivory on survival of recruit-sized *S*. *polycystum*

**Fig 3:**
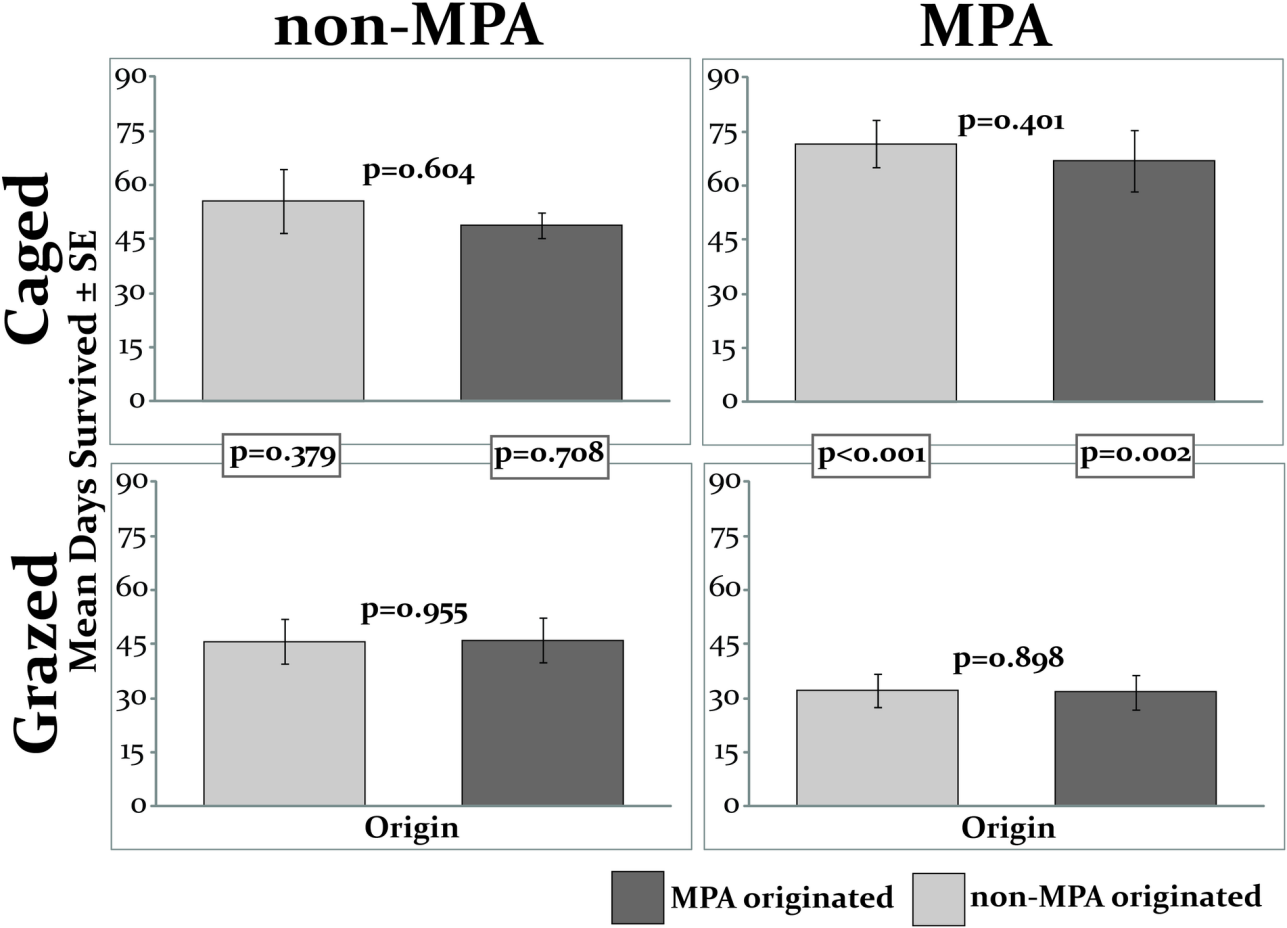
Survival of recruit-sized ramets reciprocally transplanted between MPA and non-MPA when caged or exposed. The experiment ran for 112 days; p-values above bars are from paired t-tests comparing the two origins within each treatment and location. Boxed p-values are from unpaired t-tests comparing ramets of the same origin in the caged and grazed treatments. N = 10 for each treatment and α = 0.025 to correct for multiple contrasts.

In contrast to the significant effect of origin on growth of mature *S*. *polycystum* fronds (Fig 2), origin did not affect duration of survival of recruit-sized ramets in any location (p≥0.401; paired t-tests; Fig 3).

Of the 320 initial recruit-sized ramets deployed in the reciprocal transplant experiment, 10 (3.1%) were lost (they and their basal substrate were missing). Of the remaining 310, 271 (87.4%) died or appeared to be consumed and 39 (12.6%) remained alive at the end of the 4 months. Of the 39 survivors, 25 (64.1%) were in the MPA closed-cages while none survived in the MPA open-sided cages. In the non-MPA, 7 ramets (17.9%) survived in the complete cages and 7 survived in the open cages.

When duration of survival was assessed, protection from herbivory strongly increased the duration of ramet survival in the MPA: juvenile-sized ramets protected from fish grazing survived more than twice as long as those that were unprotected in the open sided cages (p≤0.002; independent samples t-tests; Fig 3). Conversely, caging had no effect on duration of survival in the non-MPA (p≥0.379; Fig 3).

### Effect of conspecifics on survival and growth of mature fronds

**Fig 4:**
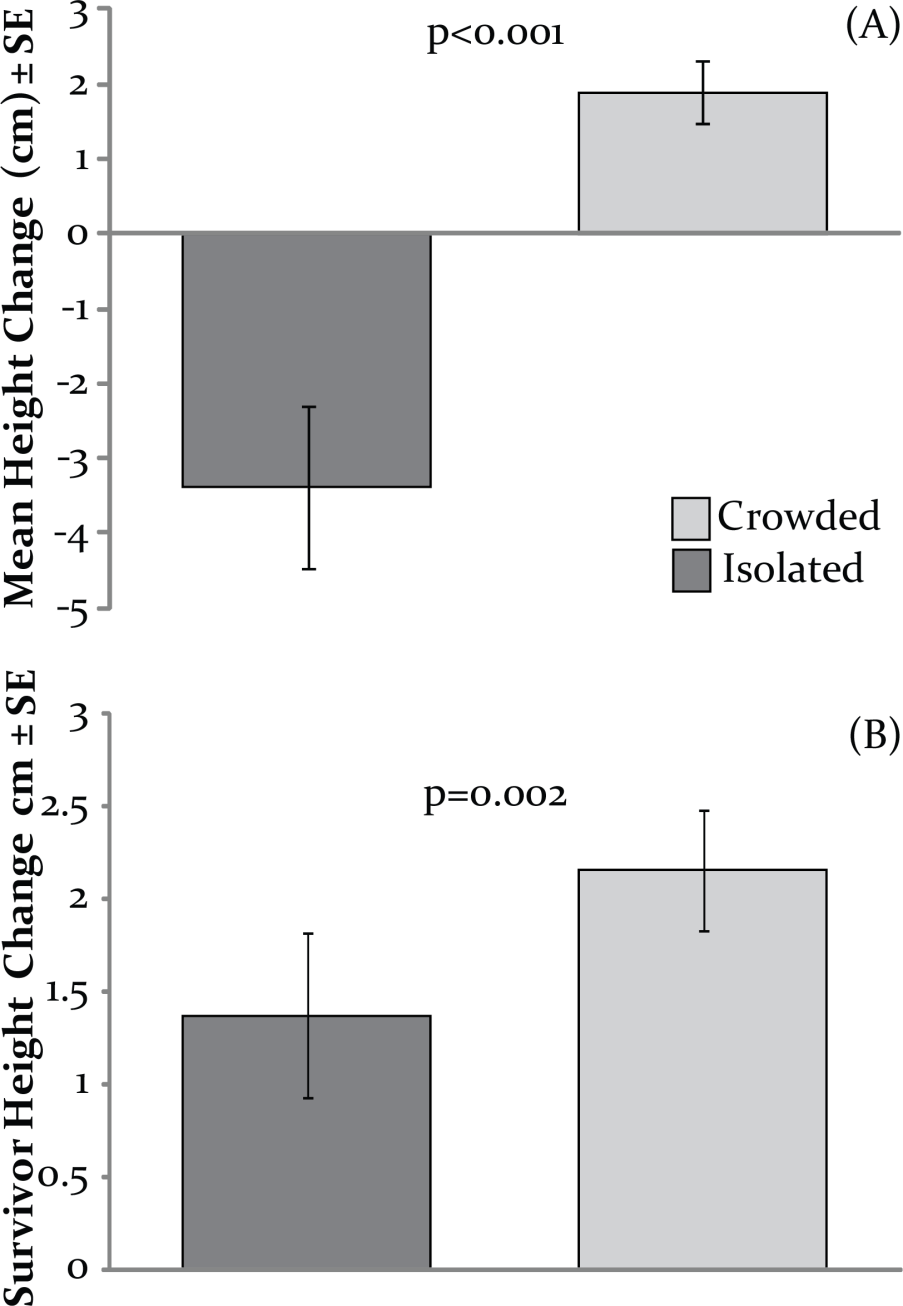
Growth ofmature *S*. *polycystum* fronds transplanted into or outside *Sargassum* beds. (A) Data for all the transplanted fronds (n = 20), presented p-value is from a Wilcoxon signed-rank test(B)Data restricted to fronds that survived the entire experiment (n = 16), presented p-value is from a paired t-test.

There was a clear difference in survival and growth of adult fronds placed into crowded versus isolated areas in Votua’s non-MPA site. We found that adult fronds experienced increased survival and growth when placed within *Sargassum* beds compared to nearby isolated areas. In the crowded condition, 78 out of 80 (97.5%) fronds remained at the end of the two week experiment; only two had been grazed (2.5%) and these were completely consumed. In the isolated treatment only 47 out of 80 (58.8%) remained while 33 (41.2%) were completely grazed. None had dislodged from the ropes as the basal stipes were still entwined within the rope strands for all fronds, including those removed by grazing. When all fronds were analysed, those in the crowded condition increased an average of ~2cm (~20%), while those in the isolated condition declined by ~3.5cm (~35%; p<0.001; n = 20; Wilcoxon signed-rank test; Fig 4A). When only the survivors were analysed, those in the crowded conditions still increased significantly more than those in the isolated conditions (crowded ~2.1cm; isolated ~1.4cm; p = 0.002; n = 16; paired t-test) indicating that even when herbivory is minimal, conditions in the *Sargassum* beds are more suitable for *S*. *polycystum* growth than conditions in isolated areas.

### Effect of conspecifics on survival and growth of recruit-sized ramets

When we transplanted recruit-sized ramets in a similar experiment into both MPAs and non-MPAs at each of two villages, once again survival and growth were higher when ramets were crowded by conspecifics. By the end of the three month experiment in Votua, 16 individuals were lost from the initial 240, leaving 224. Of these 224, 86 survived the duration of the experiment, of which 67 (78%) were in the established *Sargassum* beds (crowded condition). In Vatu-o-lailai, 15 individuals were lost leaving a total of 225. Of these 225, 116 survived of which 79 (68%) were within the algal beds.

**Fig 5:**
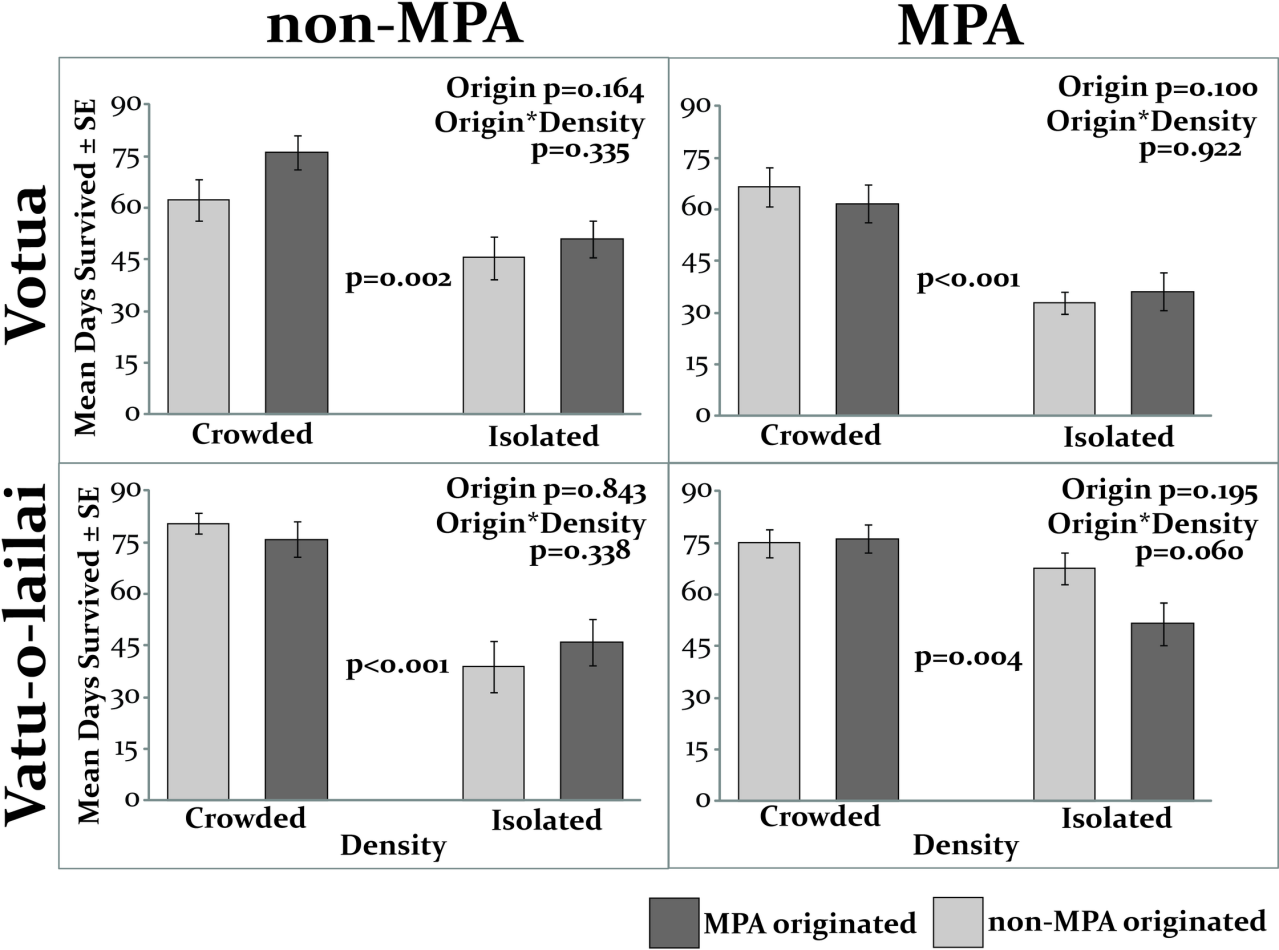
Survival of recruit-sized ramets growing in crowded or isolated densities in the MPA and non-MPA of both villages (N = 15). Initial height was subtracted from final height for all ramets, meaning those that died were included as negative values; statistical analyses were by Permutation ANOVA.

Not only was proportion of survivors higher in the crowded condition, but when survival was measured as number of days survived, ramets survived longer when crowded by conspecifics (average between 62 and 80 days) than when they were isolated (between 33 and 68 days; p≤0.004; Fig 5), and this occurred regardless of whether the experiment was conducted in the MPAs or non-MPAs.

**Fig 6:**
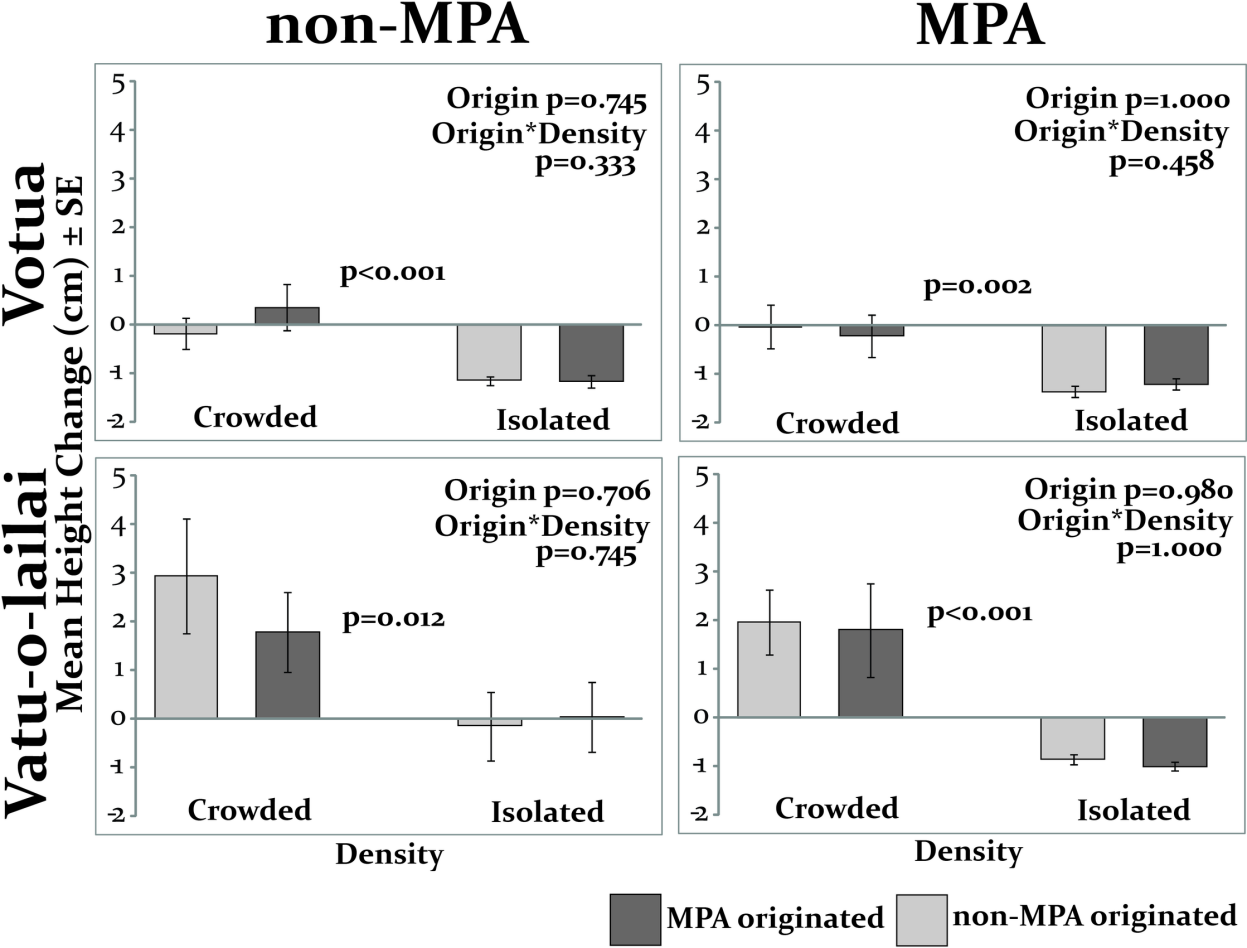
Growth of recruit-sized ramets growing in crowded or isolated densities in the MPA and non-MPA of both villages (N = 15). Initial height was subtracted from final height for all ramets, meaning those that died were included as negative values; statistical analyses were by Permutation ANOVA.

Net change in height was also significantly greater in the crowded (average height change between -0.2 and 3 cm) than in the isolated condition (-1.4 and ~0 cm; p≤0.012; Fig 6), and this occurred in both the MPA and non-MPA setting.

Similar to the previous experiment using recruit-sized ramets (Fig 3), ramet origin was not a significant factor here; neither duration of survival nor growth varied by origin in any location (Origin p≥0.100 Fig 5; Origin p≥0.706 Fig 6; permutation ANOVA). The interaction was also not significant in any location (Origin*Location p≥0.060).

Thus regardless of where the ramets originated and whether they are placed in MPA or non-MPA habitats, both survival and growth were higher when surrounded by conspecifics.

### Microsatellite analysis

The estimate of Fst was <0.05 for each individual locus (S1 Appendix) and 0.0048 over the five loci combined, indicating negligible differentiation across these four populations.

## Discussion

Dominance of *S*. *polycystum* on degraded reefs can suppress recovery to a coral-dominated state [5,6,28]. At our study sites, herbivory was the dominant process negatively affecting *S*. *polycystum* survival and growth (Fig 3), while the presence of conspecifics was the dominant positive influence (Figs 4, 5 and 6). That these factors apply to both mature and recruit-sized ramets and in habitats of vastly different community composition (intact and degraded reefs), suggest they are major factors influencing populations of *S*. *polycystum* in this region. The origin of the alga and the habitat in which it grew were of secondary importance in influencing *S*. *polycystum* survival and growth.

The common appreciation for the negative effects of intraspecific competition often obscures the fact that elevated plant density can produce positive feedbacks that more than compensate for competitive costs, especially when plants are in stressful physical or biological circumstances [36]. Just as animals achieve positive benefits by aggregating into herds, schools, or flocks [37,38], experiments are increasingly demonstrating similar positive effects of “herding” or dense aggregations in organisms as diverse as seaweeds [18], marsh plants [39], mangroves [40], oysters [41], and microbes [42]. Once *Sargassum* beds are established, they clearly suppress herbivory on juveniles and adults growing within the bed versus those a few metres outside the bed (Figs 4A and 5). However, in addition to reducing herbivory, *Sargassum* beds also appear to generate a positive physiological effect on congeners; growth of ungrazed, mature fronds placed inside *Sargassum* beds was 50% greater than growth of undgrazed fronds placed outside the bed (Fig 4B). Numerous studies on aggregated conspecifics find advantages due to protection from consumers or enhanced awareness of resource patches or dangers, but these advantages often have to counterbalance a physiological cost due to increased intraspecific competition. Here we detected a group advantage of reduced attack by consumers, but we also detected a physiological advantage, rather than cost, to intraspecific crowding. Reasons for this are unknown, but previous studies on plant aggregations have sometimes found that aggregated plants are better than individuals at lessening physical stresses such as desiccation, anaerobic soils, or erosion [36,39], resulting in a physiological benefit of aggregation.

In the habitats where we worked, light is high and turbulence and flow are often considerable, meaning that light resources may be plentiful and nutrient replacement high, which would minimise intraspecific competition. Hence, our results could be different in lower light (deeper) conditions or habitats with less flow to break down diffusion gradients. However, it is also possible that *Sargassum* crowding produces direct positive effects for members of the group. Possible hypotheses include: 1) shading within the bed reduces light shock or photorespiration in these shallow waters (e.g., [43]); 2) baffling of wave force reduces damage due to sand scour or other physical processes [44]; 3) retention of DOC or other leached metabolites within the bed enhance beneficial microbes or suppress damaging microbes and increase the net growth of *Sargassum* individuals in the group [45]; or 4) other unknown benefits generated by positive feedbacks from *Sargassum* density. Although additional work is needed to clarify the mechanism of protective benefit from conspecifics, our data indicate that established *Sargassum* beds act as a positive feedback, or stabilising mechanism [6,46], that promotes algal growth and persistence and potentially also expansion. These positive effects on *Sargassum* likely enhance the resilience of macroalgal dominated reefs and suppress recovery of the coral dominated state [1,6].

Although we saw no influence of algal origin on survival or growth of recruit-sized ramets, we did detect a significant effect of algal origin on growth of mature fronds. Mature fronds from the MPA grew significantly more than those from the non-MPA in three of our four locations (Fig 2). Isotopic analyses of macroalgae from these locations also suggested that those in the MPAs were growing more rapidly [26]. It is surprising that mature fronds exhibited an effect of origin while recruits did not. We speculate this could either be because mature fronds have accumulated nutrient stores and can rely on them post-transplant, or alternatively, because recruit-sized ramets are able to acclimatise by responding rapidly to new conditions, while mature fronds cannot. Another potential explanation is that this difference results from genetic differences or maternal effects that were not detected in the five microsatellite loci we analysed. However all Fst values from our microsatellite analysis were below 0.05 (S1 Appendix), which suggests minimal genetic differentiation [47]. At such small spatial scales, we would only expect to see genetic differences if selective pressures were strong and distinct, and we found herbivory and the presence of conspecifics to be major forces in both the MPAs and non-MPAs. Thus it is likely that *S*. *polycystum* in this region is one population that is responding phenotypically to different drivers in different locations. This is in agreement with other studies that have reported plasticity in growth rate without concomitant variation in genetics [48]. Additionally, previous research has found little genetic differentiation in this species across large spatial scales [49,50].

Although we have not addressed how *Sargassum* beds become established, we do see that they are a stabilising mechanism [46] that promotes continued *S*. *polycystum* dominance via positive feedbacks and may be preventing the recovery of coral reef communities [5,13]. It appears that removal of herbivores from the non-MPAs through extensive fishing created spatial refuges from herbivory which allowed the initial establishment of *S*. *polycystum* [22]. Once established, this species creates a positive feedback that enhances the fitness of both recruit-sized and mature conspecifics, making macroalgal dominated areas resilient, and less likely to revert back to coral domination. The ability of *S*. *polycystum* to store reserves in rhizomes that are protected within the reef structure may further enhance its resilience in the non-MPAs [51].

Our findings illuminate an interesting interplay between the different population controls operating in this system. Of the factors we addressed, herbivore escape appears to be the primary factor promoting *S*. *polycystum* survival and growth. The difference in *S*. *polycystum* abundance between the MPAs and non-MPAs is dramatic, but this does not arise from the non-MPAs being a more favourable environment since growth is lower in the non-MPAs. Instead, the combination of reduced herbivory and increased algal density in the non-MPAs act in a positive feedback manner [6] to promote persistence of *S*. *polycystum* and enhance the resilience of the degraded reef state. We detected no negative effects of intra-specific competition as growth and survival were greater in areas of high *Sargassum* density even when herbivory appeared to be minimal. This study highlights the positive influence *Sargassum* beds have on their own species and thus how they generate positive feedbacks that stabilise macroalgal dominance on reefs and suppresses recovery to coral and fish dominated systems.

## Supporting Information

S1 Appendix Details on the microsatellite analysis. (PDF)

## References

[1] Mumby PJ, Steneck RS. Coral reef management and conservation in light of rapidly evolving ecological paradigms. Trends Ecol Evol. 2008;23(10):555–63. 10.1016/j.tree.2008.06.011 18722687

[2] Hughes TP, Graham NA, Jackson JB, Mumby PJ, Steneck RS. Rising to the challenge of sustaining coral reef resilience. Trends Ecol Evol. 2010;25(11):633–42. 10.1016/j.tree.2010.07.011 20800316

[3] Alvarez-Filip L, Dulvy NK, Gill JA, Côté IM, Watkinson AR. Flattening of Caribbean coral reefs: region-wide declines in architectural complexity. Proc R Soc Lond B Biol Sci. 2009;276(1669):3019–25. 10.1098/rspb.2009.0339 PMC2817220 19515663

[4] Wilson SK, Graham NA, Fisher R, Robinson J, Nash K, Chong‐Seng K, Polunin NV, Aumeeruddy R, Quatre R. Effect of macroalgal expansion and marine protected areas on coral recovery following a climatic disturbance. Conserv Biol. 2012;26(6):995–1004. 10.1111/j.1523-1739.2012.01926.x 22971046

[5] Dixson DL, Abrego D, Hay ME. Chemically mediated behavior of recruiting corals and fishes: a tipping point that may limit reef recovery. Science. 2014;345(6199):892–7. 10.1126/science.1255057 25146281 PMC4470392

[6] Nyström M, Norström AV, Blenckner T, de la Torre-Castro M, Eklöf JS, Folke C, Österblom H, Steneck RS, Thyresson M, Troell M. Confronting feedbacks of degraded marine ecosystems. Ecosystems. 2012;15(5):695–710.

[7] Webster FJ, Babcock RC, Van Keulen M, Loneragan NR. Macroalgae inhibits larval settlement and increases recruit mortality at Ningaloo Reef, Western Australia. PloS one. 2015;10(4):e0124162 10.1371/journal.pone.0124162 25898011 PMC4405272

[8] Thurber RV, Burkepile DE, Correa AM, Thurber AR, Shantz AA, Welsh R, et al Macroalgae decrease growth and alter microbial community structure of the reef-building coral, *Porites astreoides*. PLoS One. 2012;7(9):e44246 10.1371/journal.pone.0044246 22957055 PMC3434190

[9] Foster NL, Box SJ, Mumby PJ. Competitive effects of macroalgae on the fecundity of the reef-building coral *Montastraea annularis*. Mar Ecol Prog Ser. 2008;367:143–52.

[10] Rasher DB, Hay ME. Chemically rich seaweeds poison corals when not controlled by herbivores. Proc Natl Acad Sci USA. 2010;107(21):9683–8. 10.1073/pnas.0912095107 20457927 PMC2906836

[11] Rasher DB, Stout EP, Engel S, Kubanek J, Hay ME. Macroalgal terpenes function as allelopathic agents against reef corals. Proc Natl Acad Sci USA. 2011;108(43):17726–31. 10.1073/pnas.1108628108 22006333 PMC3203809

[12] Nugues MM, Smith GW, Hooidonk RJ, Seabra MI, Bak RP. Algal contact as a trigger for coral disease. Ecol Lett. 2004;7(10):919–23.

[13] Kuffner IB, Walters LJ, Becerro MA, Paul VJ, Ritson-Williams R, Beach KS. Inhibition of coral recruitment by macroalgae and cyanobacteria. Mar Ecol Prog Ser. 2006;323:107–17.

[14] Williams ID, Polunin NV, Hendrick VJ. Limits to grazing by herbivorous fishes and the impact of low coral cover on macroalgal abundance on a coral reef in Belize. Mar Ecol Prog Ser. 2001;222:187–96.

[15] Mumby PJ, Hastings A, Edwards HJ. Thresholds and the resilience of Caribbean coral reefs. Nature. 2007;450(7166):98–101. 17972885 10.1038/nature06252

[16] Hoey AS, Bellwood DR. Suppression of herbivory by macroalgal density: a critical feedback on coral reefs?. Ecol Lett. 2011;14(3):267–73. 10.1111/j.1461-0248.2010.01581.x 21265975

[17] Van Alstyne KL, Ehlig JM, Whitman SL. Feeding preferences for juvenile and adult algae depend on algal stage and herbivore species. Mar Ecol Prog Ser. 1999;180:179–85.

[18] Hay ME. The functional morphology of turf-forming seaweeds: persistence in stressful marine habitats. Ecology. 1981; 6 1:739–50.

[19] Hoey AS. Size matters: macroalgal height influences the feeding response of coral reef herbivores. Mar Ecol Prog Ser. 2010;411:299–302.

[20] Mumby PJ, Dahlgren CP, Harborne AR, Kappel CV, Micheli F, Brumbaugh DR, Holmes et al Fishing, trophic cascades, and the process of grazing on coral reefs. Science. 2006;311(5757):98–101. 16400152 10.1126/science.1121129

[21] Burkepile DE, Hay ME. Herbivore species richness and feeding complementarity affect community structure and function on a coral reef. Proc Natl Acad Sci USA. 2008; 105(42): 16201–16206. 10.1073/pnas.0801946105 18845686 PMC2565647

[22] Rasher DB, Hoey AS, Hay ME. Consumer diversity interacts with prey defenses to drive ecosystem function. Ecology. 2013; 94(6):1347–58. 23923498 10.1890/12-0389.1 PMC3752656

[23] Conover DO, Munch SB. Sustaining fisheries yields over evolutionary time scales. Science. 2002;297(5578):94–6. 12098697 10.1126/science.1074085

[24] Stockwell CA, Hendry AP, Kinnison MT. Contemporary evolution meets conservation biology. Trends Ecol Evol. 2003;18(2):94–101.

[25] Simpson R. Assessing MPA effectiveness through observing the relative abundances of community-selected indicator populations over time. A case study of the Korolevu-i-wai qoliqoli on the Coral Coast, Fiji. MSc thesis, University of South Pacific. 2010

[26] Dell C, Montoya JP, Hay ME. Effect of marine protected areas (MPAs) on consumer diet: MPA fish feed higher in the food chain. Mar Ecol Prog Ser. 2015; 540:227. 27340314 10.3354/meps11487 PMC4913280

[27] Bonaldo RM, Hay ME. Seaweed-coral interactions: variance in seaweed allelopathy, coral susceptibility, and potential effects on coral resilience. PloS One. 2014; 9(1), e85786 10.1371/journal.pone.0085786 24465707 PMC3899053

[28] Hughes TP, Rodrigues MJ, Bellwood DR, Ceccarelli D, Hoegh-Guldberg O, McCook L, et al Phase shifts, herbivory, and the resilience of coral reefs to climate change. Curr Biol. 2007;17(4):360–5. 17291763 10.1016/j.cub.2006.12.049

[29] Mattio L, Payri CE, Verlaque M. Taxonomic revision and geographic distribution of the subgenus *Sargassum* (Fucales, Phaeophyceae) in the Western and Central Pacific Islands based on morphological and molecular analyses1. J Phycol. 2009;45(5):1213–27. 10.1111/j.1529-8817.2009.00737.x 27032365

[30] N'Yeurt AD, Iese V. The proliferating brown alga *Sargassum polycystum* in Tuvalu, South Pacific: assessment of the bloom and applications to local agriculture and sustainable energy. J App Phycol. 2014:1–9.

[31] Kendrick GA, Walker DI. Dispersal of propagules of *Sargassum* spp.(Sargassaceae: Phaeophyta): observations of local patterns of dispersal and consequences for recruitment and population structure. J Exp Mar Biol Ecol. 1995;192(2):273–88.

[32] Kinnison MT, Hendry AP. The pace of modern life II: from rates of contemporary microevolution to pattern and process In Microevolution Rate, Pattern, Process 2001; pp. 145–164. Springer Netherlands. 11838763

[33] Wheeler RE. Permutation tests for linear models in R. Available at http://cran.r-project.org/web/packages/lmPerm/vignettes/lmPerm. 2010.

[34] Wright S. The genetical structure of populations. Ann Eugen. 1951; 15:323–354. 24540312 10.1111/j.1469-1809.1949.tb02451.x

[35] Rousset F. genepop’007: a complete re‐implementation of the genepop software for Windows and Linux. Mol Ecol Resour. 2008;8(1):103–6. 10.1111/j.1471-8286.2007.01931.x 21585727

[36] He Q, Bertness MD, Altieri AH. Global shifts towards positive species interactions with increasing environmental stress. Ecol Lett. 2013;16(5):695–706. 10.1111/ele.12080 23363430

[37] Parrish JK, Edelstein-Keshet L. Complexity, pattern, and evolutionary trade-offs in animal aggregation. Science. 1999;284(5411):99–101. 10102827 10.1126/science.284.5411.99

[38] Sumpter DJ. The principles of collective animal behaviour. Philos Trans R Soc Lond B Biol Sci. 2006;361(1465):5–22. 16553306 10.1098/rstb.2005.1733 PMC1626537

[39] Silliman BR, Schrack E, He Q, Cope R, Santoni A, Van Der Heide T, et al Facilitation shifts paradigms and can amplify coastal restoration efforts. Proc Natl Acad Sci USA. 2015;112(46):14295–300. 10.1073/pnas.1515297112 26578775 PMC4655511

[40] Kumara MP, Jayatissa LP, Krauss KW, Phillips DH, Huxham M. High mangrove density enhances surface accretion, surface elevation change, and tree survival in coastal areas susceptible to sea-level rise. Oecologia. 2010;164(2):545–53. 10.1007/s00442-010-1705-2 20593198

[41] Schulte DM, Burke RP, Lipcius RN. Unprecedented restoration of a native oyster metapopulation. Science. 2009;325(5944):1124–8. 10.1126/science.1176516 19644073

[42] Darch SE, West SA, Winzer K, Diggle SP. Density-dependent fitness benefits in quorum-sensing bacterial populations. Proc Natl Acad Sci USA. 2012;109(21):8259–63. 10.1073/pnas.1118131109 22566647 PMC3361460

[43] Jompa J, McCook LJ. Seaweeds save the reef?!: Sargassum canopy decreases coral bleaching on inshore reefs. Reef Res. 1998; 8(5).

[44] Mach KJ, Tepler SK, Staaf AV, Bohnhoff JC, Denny MW. Failure by fatigue in the field: a model of fatigue breakage for the macroalga *Mazzaella*, with validation. J Exp Mar Biol Ecol. 2011; 214(9):1571–1585. 10.1242/jeb.051623 21490265

[45] Singh RP, Reddy CRK. Unraveling the functions of the macroalgal microbiome. Front Microb. 2015; 6:1433. 10.3389/fmicb.2015.01488 PMC4700259 26779144

[46] Fong P, Paul VJ. Coral reef algae In: Dubinsky Z, Stambler N editors. Coral reefs: an ecosystem in transition. Springer Science & Business Media; 2011;pp 241–272.

[47] Weir BS Genetic Data Analysis 2: Methods for Discrete Population Genetic Data. Sinauer Associates, Inc. Publishers; 1996.

[48] Trtikova M, Edwards PJ, Güsewell S. No adaptation to altitude in the invasive plant Erigeron annuus in the Swiss Alps. Ecography. 2010;33(3):556–64.

[49] Chan SW, Cheang CC, Chirapart A, Gerung G, Tharith C, Ang P. Homogeneous population of the brown alga *Sargassum polycystum* in Southeast Asia: possible role of recent expansion and asexual propagation. PloS one. 2013;8(10):e77662 10.1371/journal.pone.0077662 24147050 PMC3798308

[50] Kantachumpoo A, Uwai S, Noiraksar T, Komatsu T. Levels and distribution patterns of mitochondrial cox3 gene variation in brown seaweed, *Sargassum polycystum* C. Agardh (Fucales, Phaeophyceae) from Southeast Asia. J App Phycol. 2014;26(2):1301–8.

[51] Grime JP, Hunt R. Relative growth-rate: its range and adaptive significance in a local flora. J Ecol. 1975; 7 1:393–422.

