# Supplementary material for: Positive Feedbacks Enhance Macroalgal Resilience on Degraded Coral Reefs
Source: PLoS One. 2016 May 17;11(5):e0155049. doi: 10.1371/journal.pone.0155049 (PMC4871466; doi:10.1371/journal.pone.0155049)
Supplement: S1 Appendix — (PDF) [file pone.0155049.s001.pdf]

SUPPLEMENTARY INFO

Sample Collection for Microsatellite Analysis

In May 2013, the uppermost three leaves of 50 *S. polycystum* ramets were collected from the MPAs and non-MPAs in Votua and Vatu-o-lailai (Fig. 1), maintaining a minimum distance of 3 metres between each sample to reduce the possibility of sampling clones. Samples were collected a minimum of ~40m from the boundaries of the MPA and non-MPA borders to avoid edge effects. To minimize potential differences in physical conditions, collecting sites in the MPA and non-MPA areas were chosen to have comparable depth and distance from shore. Samples were shaken to remove particulates and preserved in molecular grade ethanol, which was replaced after the first 48 hours.

DNA Extraction, Amplification & Analysis

DNA was isolated from 2-3mm^2 of between 29-43 *S. polycystum* samples per site using DNeasy Blood & Tissue Kit (Qiagen, The Netherlands) and purified with DNA, RNA & Protein Purification kit (Macherey-Nagel, Germany). All polymerase chain reactions (PCR) were run on an Eppendorf AG Thermal Cycler with a total volume of 10μL which contained: 6.8 μL DI water; 1 μL 10x PCR buffer; 1μL diluted DNA; 0.2μL of each dNTP, forward primer, reverse primer and GO Taq polymerase buffer (5u/μL Promega) and 0.4 μL $MgCl_2$ (25mM, Thermo Scientific; except primer 38 which contained 0.8μL $MgCl_2$). Primer 9 was amplified with the following profile: initial denaturation at 95°C for 2 minutes; 45 cycles of 95°C for 30 seconds, 48°C for 1 minute and 72°C for 1 minute; followed by a final extension at 72°C for 6 minutes. All other primers were amplified with the following profile: denaturation at 95°C for 2 minutes, followed by 40 cycles of 95°C for 30 seconds, 50°C for 1 minute and 72°C for 1 minute. PCR products were analysed by Nevada Genomics (Applied

Biosystems Prism 3730 DNA Analyser; University of Nevada, Reno) using GeneScan 500 LIZ size standard (Applied Biosystems, USA) and read using Peak Scanner Software 2 (Applied Biosystems, USA).

<u>Table S1: Microsatellite Primer Sequences:</u> Sequences and Fst values for the five microsatellite loci. Fst was not estimable for locus 42 as there was no allelic variation at that locus.

Primer	Forward Sequence	Reverse Sequence	Fst
1	AGG CAA GCA ACA AAC GAG TT	CAG GAT TGC AAC CAT ACC CG	0.049
9	AGGACGGGAAAAGGGAATAG	AGTTTCGGAAAGCGTTCTCA	-0.01
24	ATG GGC AGT GGG TAG ACA AT	GAT TGG TTT GAC AGA GCC GG	0.002
38	CCA ACA ACC ACT GAT GTC CC	ACC CGG CTC TGT CAA ACT AA	0.001
42	CAA CTC GCC CTG TCA AAC TA	TAG TCG TCA CCC TTT CCG G	-
